# 2,6-Diisopropyl­anilinium chloride

**DOI:** 10.1107/S160053680801790X

**Published:** 2008-07-12

**Authors:** Ivan Samardjiev, Brian Samas

**Affiliations:** a Pharmaceutical Sciences, Pfizer Global R&D, Eastern Point Road, Groton, CT 06340, USA

## Abstract

The title compound, C_12_H_20_N^+^·Cl^−^, crystallizes with the chloride anions situated on twofold axes, while the cation is on a general position. All conventional hydrogen-bond donors and acceptors are utilized, forming a hydrogen-bonded ladder motif along the *c* axis. Investigation of the torsion angles between aromatic systems and isopropyl groups reveals unusual geometrical features. One isopropyl groups exhibits an expected eclipsed conformation with respect to the aromatic ring. The other isopropyl group shows a slight twist with respect to the aromatic ring. The short Cl⋯HC(methine) contact (2.88 Å) observed in the asymmetric unit is the probable reason for the twist feature around the isopropyl area.

## Related literature

For the structure of the tetra­hydro­furan solvate of the title salt, see: Bond & Doyle (2003 ▶).
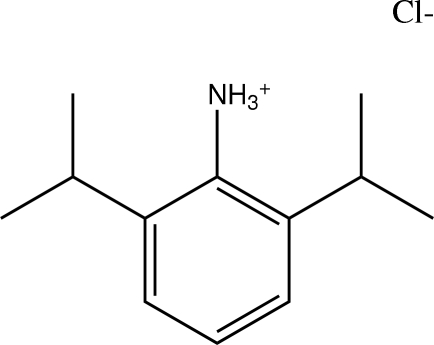


## Experimental

### 

#### Crystal data

C_12_H_20_N^+^·Cl^−^
*M* *_r_* = 213.74
Orthorhombic, 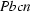

*a* = 13.0390 (3) Å
*b* = 21.0436 (4) Å
*c* = 8.9968 (2) Å
*V* = 2468.61 (9) Å^3^
*Z* = 8
Cu *K*α radiation
μ = 2.43 mm^−1^
*T* = 173 (2) K
0.36 × 0.23 × 0.21 mm

#### Data collection

Bruker SMART APEXII CCD diffractometer
Absorption correction: multi-scan (*SADABS*; Sheldrick, 2002 ▶) *T*_min_ = 0.451, *T*_max_ = 0.597
23541 measured reflections
2343 independent reflections
2248 reflections with *I* > 2σ(*I*)
*R*_int_ = 0.028

#### Refinement

*R*[*F* ^2^ > 2σ(*F* ^2^)] = 0.037
*wR*(*F* ^2^) = 0.112
*S* = 1.00
2343 reflections
144 parameters
H atoms treated by a mixture of independent and constrained refinement
Δρ_max_ = 0.35 e Å^−3^
Δρ_min_ = −0.21 e Å^−3^

### 

Data collection: *SMART* (Bruker, 2006 ▶; cell refinement: *SAINT* (Bruker, 2006 ▶); data reduction: *SAINT*; program(s) used to solve structure: *SHELXS97* (Sheldrick, 2008 ▶); program(s) used to refine structure: *SHELXL97* (Sheldrick, 2008 ▶); molecular graphics: *SHELXTL* (Sheldrick, 2008 ▶); software used to prepare material for publication: *SHELXTL*.

## Supplementary Material

Crystal structure: contains datablocks I, global. DOI: 10.1107/S160053680801790X/bh2172sup1.cif

Structure factors: contains datablocks I. DOI: 10.1107/S160053680801790X/bh2172Isup2.hkl

Additional supplementary materials:  crystallographic information; 3D view; checkCIF report

## Figures and Tables

**Table 1:** Hydrogen-bond geometry (Å, °)

*D*—H⋯*A*	*D*—H	H⋯*A*	*D*⋯*A*	*D*—H⋯*A*
N13—H13*X*⋯Cl1*X*^i^	0.90 (2)	2.54 (2)	3.3777 (12)	154.7 (15)
N13—H13*Y*⋯Cl1*Y*	0.93 (2)	2.16 (2)	3.0753 (12)	167.2 (16)
N13—H13*Z*⋯Cl1*X*	0.921 (19)	2.352 (19)	3.2493 (12)	164.8 (14)

Symmetry code: (i) 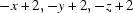.

**Fig. 1.**
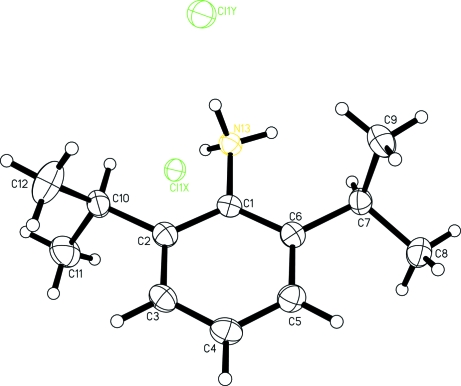
View of the constituents of (I), showing the atom-labeling scheme and displacement ellipsoids drawn at the 30% probability level. H atoms are represented by circles of arbitrary size.

**Fig. 2.**
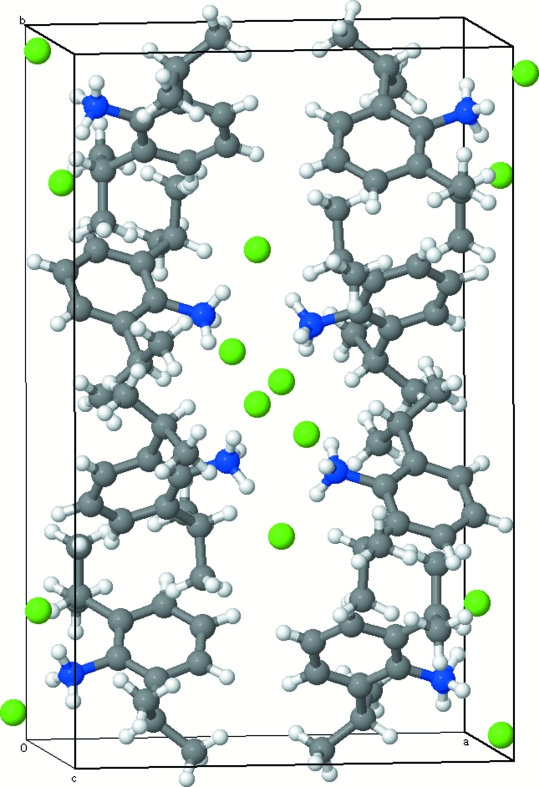
The crystal structure viewed along the [001] direction, showing four Cl- anions spaced between four DIPA+ cations.

## supplementary crystallographic information

### Comment

2,6-DIPA chloride is frequently used as a starting material for pharmaceutical synthesis. Hydrochloric acid is a desirable acid for salt formation and during our efforts to purify DIPA by salt formation and crystallization, we formed the title 1:1 salt (Fig. 1). Chlorine anions have an interesting 50:50 occupancy positions, sitting on 2-fold axis with *x*,*y*,*z* coordinates as follows: Cl1X (0, *y*, 1/4) and Cl1Y (0, *y*, 3/4).

The structure has all conventional hydrogen-bond donors used. In the crystal structure, four of the protonated NH_3_ groups face the counter-ions sitting on above mentioned special positions, forming two-dimensional sheets parallel to the (010) planes (Fig. 2). Hydrogen-bond network confirms the following interactions between 50% populated Cl^-^ anions and polar ends of DIPAH^+^ (NH_3_^+^ groups): Cl1X participates a hydrogen-bonding interaction with H13X (2.54 Å separation), H13*Z* (2.35 Å), and H13Y (2.16 Å) are hydrogen-bonded to Cl1X and Cl1Y, respectively. There is an additional short contact within van der Waals radii between Cl1X and H7 (2.88 Å) yet this interaction does not occur with the Cl1Y occupancy.

The torsion angles from the aromatic group to the isopropyl groups are interesting. The expectation is that the isopropyl groups would eclipse the aromatic ring plane so that the C7—H7 and C10—H10 bonds lie in the same plane as atoms N13, C1, C6 and C7. One of these groups exhibits such a conformation (C1—C2—C10—H10 = -0.21°), while the other shows a slight twist (C1—C6—C7—H7 = -34.62°). The above mentioned Cl1X···H7 short contact is the probable reason for the obvious twist event around C1—C6—C7—H7 area. Similar events are observed in a related DIPA chloride salt structure, where only one of isopropyl hydrogen experiences van der Waals contacts with a Cl^-^ anion (Bond & Doyle, 2003).

### Experimental

A stock solution of DIPA was made in 2-propanol (85 mg, 2 ml). To a crystallizer vessel, 0.43 ml of stock solution was added with 1 equivalent of concentrated hydrochloric acid. For salt formation participation we gradually added 6 ml of methyl *t*-butyl ether, then the sample was purged with dry nitrogen for evaporation until dryness, allowed to evaporate over 24 h mark. A crystal of the title salt was removed from the crystallizer vessel and mounted on a MiTeGen loop with Paratone-N oil.

### Refinement

H atoms bonded to C atoms were placed in idealized positions and refined using a riding model with C—H = 0.93 Å for C*sp*^2^—H, 0.96 Å for CH_3_, and 0.98 Å for CH. *U*_iso_(H) values were fixed to 0.08 Å^2^. H atoms bound to N3 were located in a difference maps and their positions and isotropic displacement parameters were refined freely.

### Crystal data

**Table:**

C_12_H_20_N^+^·Cl^–^	*F*_000_ = 928
*M**_r_* = 213.74	*D*_x_ = 1.150 Mg m^−^^3^
Orthorhombic, *P**b**c**n*	Cu *K*α radiation λ = 1.54178 Å
Hall symbol: -P 2n 2ab	Cell parameters from 9259 reflections
*a* = 13.0390 (3) Å	θ = 4.0–70.8º
*b* = 21.0436 (4) Å	µ = 2.43 mm^−^^1^
*c* = 8.9968 (2) Å	*T* = 173 (2) K
*V* = 2468.61 (9) Å^3^	Prism, colourless
*Z* = 8	0.36 × 0.23 × 0.21 mm

### Data collection

**Table:**

Bruker SMART APEXII CCD diffractometer	2343 independent reflections
Radiation source: Rotating Anode	2248 reflections with *I* > 2σ(*I*)
Monochromator: Montel Multilayer Optics	*R*_int_ = 0.028
*T* = 173(2) K	θ_max_ = 71.7º
φ and ω scans	θ_min_ = 4.0º
Absorption correction: multi-scan(SADABS; Sheldrick, 2002)	*h* = −15→15
*T*_min_ = 0.451, *T*_max_ = 0.597	*k* = −25→20
23541 measured reflections	*l* = −10→9

### Refinement

**Table:**

Refinement on *F*^2^	Secondary atom site location: difference Fourier map
Least-squares matrix: full	Hydrogen site location: inferred from neighbouring sites
*R*[*F*^2^ > 2σ(*F*^2^)] = 0.037	H atoms treated by a mixture of independent and constrained refinement
*wR*(*F*^2^) = 0.112	*w* = 1/[σ^2^(*F*_o_^2^) + (0.0758*P*)^2^ + 1.0624*P*] where *P* = (*F*_o_^2^ + 2*F*_c_^2^)/3
*S* = 1.00	(Δ/σ)_max_ = 0.029
2343 reflections	Δρ_max_ = 0.35 e Å^−^^3^
144 parameters	Δρ_min_ = −0.21 e Å^−^^3^
Primary atom site location: structure-invariant direct methods	Extinction correction: none

### Fractional atomic coordinates and isotropic or equivalent isotropic displacement parameters (Å^2^)

**Table:**

	*x*	*y*	*z*	*U*_iso_*/*U*_eq_	
C1	0.79245 (10)	0.88741 (6)	0.98360 (14)	0.0207 (3)	
C2	0.77502 (10)	0.84000 (6)	1.08919 (15)	0.0238 (3)	
C3	0.67345 (11)	0.82064 (6)	1.11109 (16)	0.0276 (3)	
H3	0.6592	0.7891	1.1806	0.080*	
C4	0.59411 (11)	0.84748 (7)	1.03134 (17)	0.0296 (3)	
H4	0.5271	0.8341	1.0476	0.080*	
C5	0.61414 (10)	0.89426 (7)	0.92708 (16)	0.0276 (3)	
H5	0.5601	0.9119	0.8738	0.080*	
C6	0.71389 (11)	0.91548 (6)	0.90040 (15)	0.0229 (3)	
C7	0.73500 (11)	0.96575 (6)	0.78298 (16)	0.0250 (3)	
H7	0.7931	0.9913	0.8179	0.080*	
C8	0.64526 (13)	1.01091 (8)	0.75817 (17)	0.0356 (4)	
H8A	0.5916	0.9891	0.7059	0.080*	
H8B	0.6678	1.0467	0.7005	0.080*	
H8C	0.6199	1.0254	0.8524	0.080*	
C9	0.76786 (13)	0.93367 (8)	0.63763 (16)	0.0357 (4)	
H9A	0.8242	0.9054	0.6568	0.080*	
H9B	0.7887	0.9655	0.5674	0.080*	
H9C	0.7113	0.9100	0.5977	0.080*	
C10	0.85921 (11)	0.80846 (6)	1.17924 (16)	0.0276 (3)	
H10	0.9249	0.8271	1.1493	0.080*	
C11	0.84443 (14)	0.82083 (9)	1.34518 (18)	0.0418 (4)	
H11A	0.8456	0.8658	1.3635	0.080*	
H11B	0.8987	0.8008	1.3999	0.080*	
H11C	0.7797	0.8037	1.3764	0.080*	
C12	0.86270 (15)	0.73722 (8)	1.1448 (2)	0.0491 (5)	
H12A	0.7992	0.7179	1.1742	0.080*	
H12B	0.9182	0.7180	1.1985	0.080*	
H12C	0.8729	0.7311	1.0401	0.080*	
N13	0.89957 (8)	0.90795 (6)	0.95727 (13)	0.0218 (3)	
H13X	0.9045 (14)	0.9424 (9)	0.899 (2)	0.034 (5)*	
H13Y	0.9357 (15)	0.8761 (9)	0.908 (2)	0.036 (4)*	
H13Z	0.9318 (14)	0.9187 (8)	1.045 (2)	0.030 (4)*	
Cl1X	1.0000	0.97357 (2)	1.2500	0.02472 (17)	
Cl1Y	1.0000	0.80980 (2)	0.7500	0.03006 (18)	

### Atomic displacement parameters (Å^2^)

**Table:**

	*U*^11^	*U*^22^	*U*^33^	*U*^12^	*U*^13^	*U*^23^
C1	0.0213 (6)	0.0208 (6)	0.0200 (6)	−0.0023 (4)	0.0011 (5)	−0.0029 (5)
C2	0.0270 (7)	0.0224 (6)	0.0221 (7)	−0.0008 (5)	0.0012 (5)	−0.0014 (5)
C3	0.0306 (7)	0.0243 (6)	0.0279 (7)	−0.0061 (5)	0.0033 (6)	0.0016 (5)
C4	0.0244 (7)	0.0315 (7)	0.0329 (8)	−0.0085 (5)	0.0015 (6)	−0.0022 (6)
C5	0.0238 (7)	0.0307 (7)	0.0282 (7)	−0.0025 (5)	−0.0039 (5)	−0.0015 (6)
C6	0.0255 (7)	0.0229 (6)	0.0204 (7)	−0.0018 (5)	−0.0013 (5)	−0.0023 (5)
C7	0.0247 (7)	0.0267 (7)	0.0237 (6)	−0.0012 (5)	−0.0028 (5)	0.0032 (5)
C8	0.0318 (8)	0.0357 (8)	0.0392 (9)	0.0041 (7)	−0.0018 (6)	0.0106 (6)
C9	0.0462 (9)	0.0374 (8)	0.0235 (7)	−0.0010 (7)	0.0013 (6)	0.0029 (6)
C10	0.0296 (7)	0.0275 (7)	0.0258 (7)	−0.0001 (5)	−0.0005 (6)	0.0059 (5)
C11	0.0436 (9)	0.0560 (10)	0.0259 (8)	−0.0009 (7)	−0.0025 (7)	0.0044 (7)
C12	0.0539 (11)	0.0319 (8)	0.0614 (12)	0.0133 (7)	−0.0163 (9)	−0.0029 (8)
N13	0.0217 (6)	0.0230 (6)	0.0208 (6)	−0.0015 (4)	0.0001 (4)	0.0008 (5)
Cl1X	0.0248 (3)	0.0249 (3)	0.0244 (3)	0.000	−0.00197 (15)	0.000
Cl1Y	0.0339 (3)	0.0247 (3)	0.0315 (3)	0.000	0.00491 (17)	0.000

### Geometric parameters (Å, °)

**Table:**

C1—C2	1.3962 (18)	C8—H8C	0.9600
C1—C6	1.3994 (19)	C9—H9A	0.9600
C1—N13	1.4812 (16)	C9—H9B	0.9600
C2—C3	1.3996 (19)	C9—H9C	0.9600
C2—C10	1.5171 (19)	C10—C11	1.528 (2)
C3—C4	1.380 (2)	C10—C12	1.532 (2)
C3—H3	0.9300	C10—H10	0.9800
C4—C5	1.385 (2)	C11—H11A	0.9600
C4—H4	0.9300	C11—H11B	0.9600
C5—C6	1.3959 (19)	C11—H11C	0.9600
C5—H5	0.9300	C12—H12A	0.9600
C6—C7	1.5202 (19)	C12—H12B	0.9600
C7—C8	1.524 (2)	C12—H12C	0.9600
C7—C9	1.533 (2)	N13—H13X	0.90 (2)
C7—H7	0.9800	N13—H13Y	0.93 (2)
C8—H8A	0.9600	N13—H13Z	0.921 (19)
C8—H8B	0.9600		
			
C2—C1—C6	123.12 (12)	C7—C9—H9A	109.5
C2—C1—N13	118.08 (11)	C7—C9—H9B	109.5
C6—C1—N13	118.79 (11)	H9A—C9—H9B	109.5
C1—C2—C3	117.24 (12)	C7—C9—H9C	109.5
C1—C2—C10	123.93 (12)	H9A—C9—H9C	109.5
C3—C2—C10	118.83 (12)	H9B—C9—H9C	109.5
C4—C3—C2	121.14 (13)	C2—C10—C11	110.86 (12)
C4—C3—H3	119.4	C2—C10—C12	109.97 (12)
C2—C3—H3	119.4	C11—C10—C12	111.61 (14)
C3—C4—C5	120.12 (13)	C2—C10—H10	108.1
C3—C4—H4	119.9	C11—C10—H10	108.1
C5—C4—H4	119.9	C12—C10—H10	108.1
C4—C5—C6	121.31 (13)	C10—C11—H11A	109.5
C4—C5—H5	119.3	C10—C11—H11B	109.5
C6—C5—H5	119.3	H11A—C11—H11B	109.5
C5—C6—C1	117.07 (12)	C10—C11—H11C	109.5
C5—C6—C7	120.72 (12)	H11A—C11—H11C	109.5
C1—C6—C7	122.20 (12)	H11B—C11—H11C	109.5
C6—C7—C8	113.37 (12)	C10—C12—H12A	109.5
C6—C7—C9	109.69 (11)	C10—C12—H12B	109.5
C8—C7—C9	111.36 (12)	H12A—C12—H12B	109.5
C6—C7—H7	107.4	C10—C12—H12C	109.5
C8—C7—H7	107.4	H12A—C12—H12C	109.5
C9—C7—H7	107.4	H12B—C12—H12C	109.5
C7—C8—H8A	109.5	C1—N13—H13X	113.4 (11)
C7—C8—H8B	109.5	C1—N13—H13Y	110.0 (12)
H8A—C8—H8B	109.5	H13X—N13—H13Y	105.1 (16)
C7—C8—H8C	109.5	C1—N13—H13Z	111.5 (11)
H8A—C8—H8C	109.5	H13X—N13—H13Z	105.7 (15)
H8B—C8—H8C	109.5	H13Y—N13—H13Z	110.9 (16)
			
C6—C1—C2—C3	−0.23 (19)	N13—C1—C6—C5	179.19 (11)
N13—C1—C2—C3	−179.18 (11)	C2—C1—C6—C7	−178.28 (12)
C6—C1—C2—C10	179.28 (12)	N13—C1—C6—C7	0.66 (18)
N13—C1—C2—C10	0.33 (19)	C5—C6—C7—C8	28.51 (18)
C1—C2—C3—C4	0.0 (2)	C1—C6—C7—C8	−153.01 (13)
C10—C2—C3—C4	−179.55 (13)	C5—C6—C7—C9	−96.67 (15)
C2—C3—C4—C5	0.2 (2)	C1—C6—C7—C9	81.80 (16)
C3—C4—C5—C6	−0.2 (2)	C1—C2—C10—C11	118.08 (15)
C4—C5—C6—C1	0.0 (2)	C3—C2—C10—C11	−62.42 (16)
C4—C5—C6—C7	178.53 (13)	C1—C2—C10—C12	−118.01 (16)
C2—C1—C6—C5	0.25 (19)	C3—C2—C10—C12	61.49 (18)

### Hydrogen-bond geometry (Å, °)

**Table:**

*D*—H···*A*	*D*—H	H···*A*	*D*···*A*	*D*—H···*A*
N13—H13X···Cl1X^i^	0.90 (2)	2.54 (2)	3.3777 (12)	154.7 (15)
N13—H13Y···Cl1Y	0.93 (2)	2.16 (2)	3.0753 (12)	167.2 (16)
N13—H13Z···Cl1X	0.921 (19)	2.352 (19)	3.2493 (12)	164.8 (14)

Symmetry codes: (i) −*x*+2, −*y*+2, −*z*+2.
